# Supplementary material for: A Dynamic Culture Method to Produce Ovarian Cancer Spheroids under Physiologically-Relevant Shear Stress
Source: Cells. 2018 Dec 19;7(12):277. doi: 10.3390/cells7120277 (PMC6316168; doi:10.3390/cells7120277)
Supplement: Supplementary file 1 [file cells-07-00277-s001.zip › cells-400561-supplementary/cells-400561 Supplementary materials/cells-400561 Supplementary.pdf]

cells

Supplemental Table S1: Well Parameters for CFD Simulations

Plate Type	Well Diameter (cm)	Volume (mL)	Media Height (cm)	Total Height (cm)
96 WP	0.8	0.2	0.398	1.2
48 WP	1.1	0.5	0.525	1.8
24 WP	1.8	1	0.393	1.8
12 WP	2.5	1.5	0.306	1.8
6 WP	3.5	3	0.312	1.8
Petri Dish	5	5	0.255	1.3
Petri Dish	10	15	0.191	1.3

Supplemental Figure S1

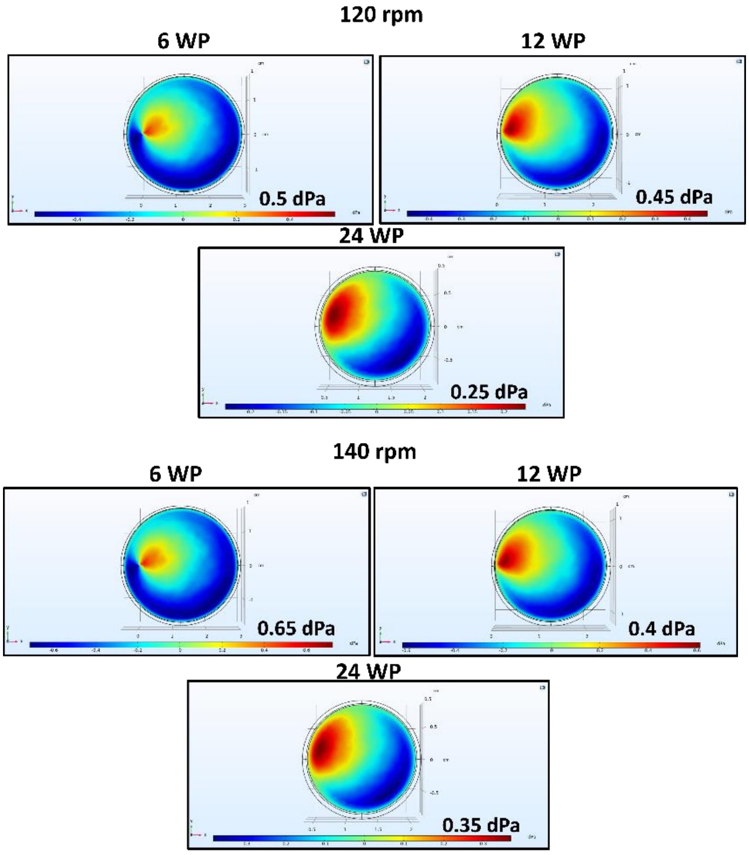

Supplemental Figure S1: COMSOL Simulations of FSS in WP at 120 and 140 rpm. Simulations using the CFD module show the tangential shear stress for 6, 12 and 24 WP and how it has increased with greater rotation speed. The maximum FSS values are reported.

Supplemental Figure S2

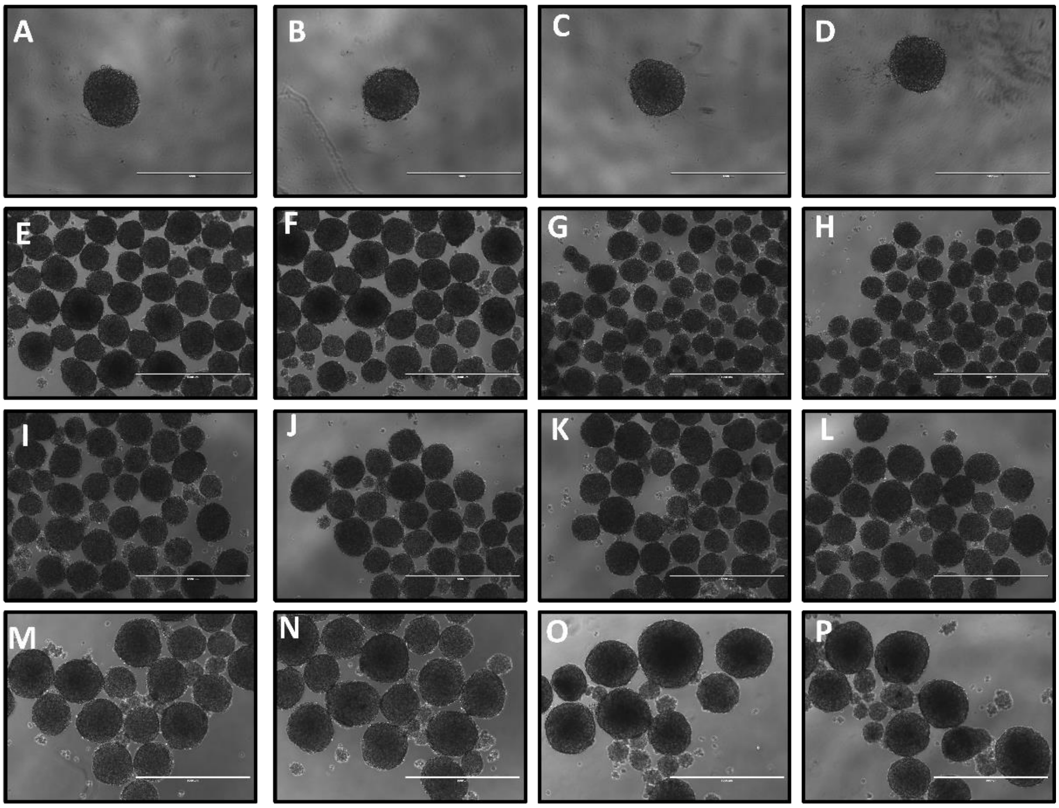

Supplemental Figure 2: Additional Representative Images of ES-2 Shaker Spheroids. All cultures were for 72 hrs, and shaker speed was 120 rpm. **A-D)** Control spheroids from round-bottom 96 WP at 1000 cells/well. Shaker spheroids were formed at 100,000 **(E-H)** 50,000 **(I-L) and** 25,000 **(M-P)** cells/mL. Scale bar: 1000 μm.

Supplemental Figure S3

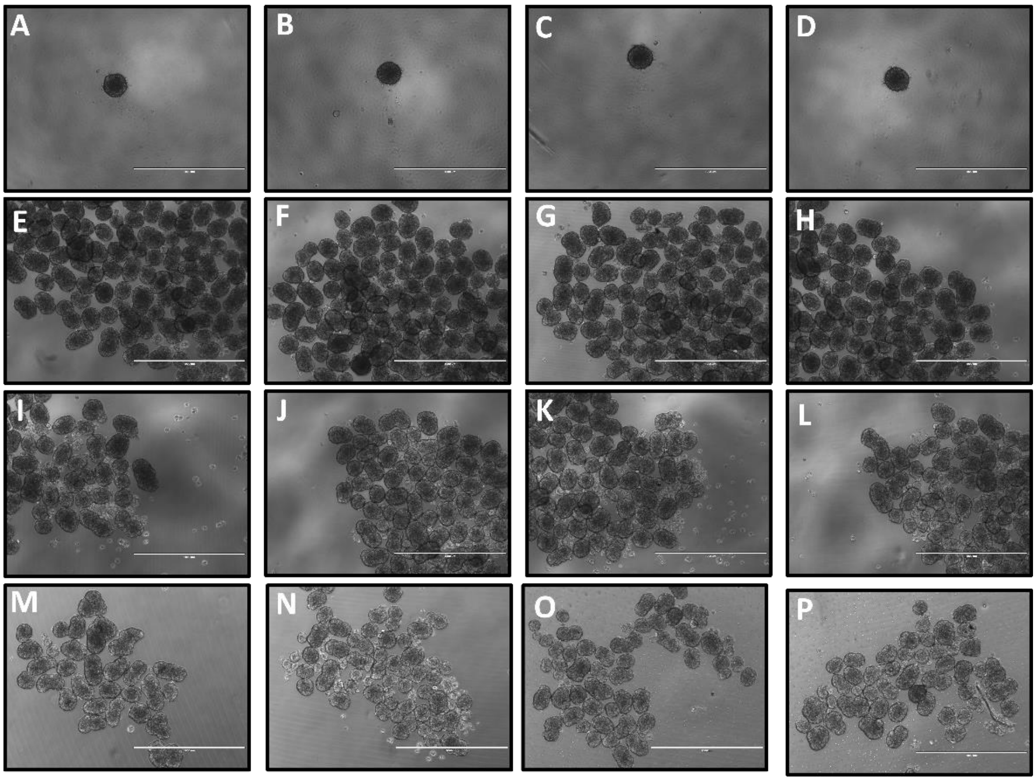

Supplemental Figure S3: Additional Representative Images of OVCA420 Shaker Spheroids. All cultures were for 72 hrs, and shaker speed was 140 rpm. **A-D)** Control spheroids from round-bottom 96 WP at 2000 cells/well. Shaker spheroids were formed at 100,000 **(E-H)** 50,000 **(I-L)** and 25,000 **(M-P)** cells/mL. Scale bar: 1000 μm.

Supplemental Figure S4

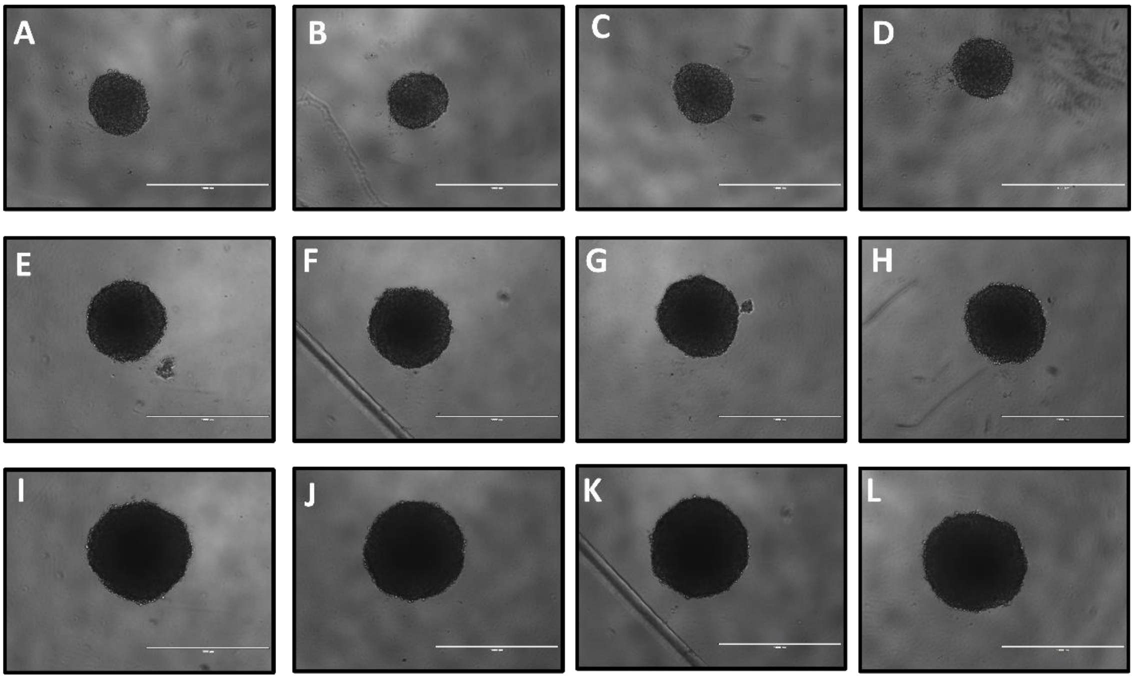

Supplemental Figure S4: Additional Representative Images of ES-2 round-bottom 96 WP Spheroids in Long-Term Culture. All cell densities were 1000 cells/well. Images were taken at 72 **(A-D)** 120 **(E-H)** and 168 **(I-L)** hrs. Scale bar: 1000 μm.

Supplemental Figure S5

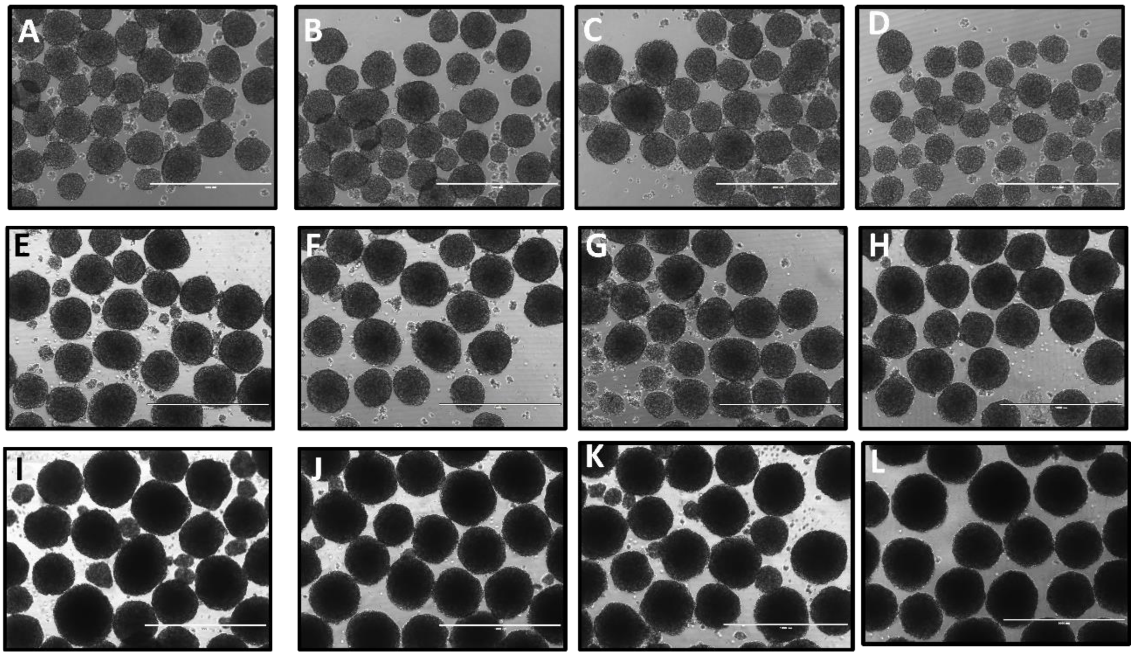

Supplemental Figure S5: Additional Representative Images of ES-2 Shaker Spheroids in Long-Term Culture. All cell densities were 100,000 cells/mL and shaker speed was 140 rpm. Images were taken at 72 **(A-D)** 120 **(E-H)** and 168 **(I-L)** hrs. Scale bar: 1000 μm.
